# Supplementary material for: MEME-LaB: motif analysis in clusters
Source: Bioinformatics. 2013 May 14;29(13):1696–7. doi: 10.1093/bioinformatics/btt248 (PMC3694638; doi:10.1093/bioinformatics/btt248)
Supplement: Supplementary Data [file supp_btt248_Supplementary_Data.zip › Supplementary_Data/Results_Files/which_gene_ids/cluster19_motif1.html]

Cluster 19 Motif 1


Cluster 19 Motif 1 is found in the following promoters

| Gene ID | Strand | Position\* |
| --- | --- | --- |

|  |  |  |
| --- | --- | --- |
| AT1G01050 | - | 269/500 |
| AT1G01160 | + | 305/500 |
| AT1G02110 | - | 486/500 |
| AT1G02180 | - | 455/500 |
| AT1G03400 | - | 92/500 |
| AT1G03687 | + | 157/500 |
| AT1G03970 | + | 332/500 |
| AT1G04120 | - | 206/500 |
| AT1G04900 | + | 224/500 |
| AT1G04910 | + | 239/500 |
| AT1G05720 | - | 12/500 |
| AT1G06110 | - | 353/500 |
| AT1G06440 | - | 67/500 |
| AT1G06690 | + | 322/500 |
| AT1G06730 | + | 444/500 |
| AT1G08030 | + | 418/500 |
| AT1G08040 | - | 391/500 |
| AT1G08710 | + | 267/500 |
| AT1G09010 | - | 1/500 |
| AT1G09060 | + | 438/500 |
| AT1G09280 | - | 203/500 |
| AT1G09300 | - | 1/500 |
| AT1G09320 | - | 158/500 |
| AT1G09620 | + | 307/500 |
| AT1G09660 | + | 293/500 |
| AT1G09710 | + | 380/500 |
| AT1G09910 | + | 66/500 |
| AT1G10310 | + | 159/500 |
| AT1G10670 | + | 74/500 |
| AT1G10870 | - | 314/500 |
| AT1G11090 | + | 389/500 |
| AT1G11930 | + | 172/500 |
| AT1G12230 | - | 286/500 |
| AT1G12530 | + | 471/500 |
| AT1G13440 | + | 201/500 |
| AT1G13560 | + | 463/500 |
| AT1G13690 | + | 487/500 |
| AT1G14850 | - | 287/500 |
| AT1G15220 | - | 252/500 |
| AT1G15280 | + | 4/500 |
| AT1G15390 | + | 396/500 |
| AT1G16570 | - | 371/500 |
| AT1G16590 | + | 422/500 |
| AT1G16710 | + | 306/500 |
| AT1G16780 | - | 20/500 |
| AT1G17760 | + | 365/500 |
| AT1G18040 | - | 391/500 |
| AT1G18700 | - | 363/500 |
| AT1G19800 | - | 446/500 |
| AT1G21280 | - | 443/500 |
| AT1G21710 | - | 163/500 |
| AT1G22860 | + | 286/500 |
| AT1G23310 | + | 310/500 |
| AT1G23890 | - | 189/500 |
| AT1G24290 | - | 267/500 |
| AT1G24793 | + | 90/500 |
| AT1G25580 | - | 193/500 |
| AT1G26370 | - | 471/500 |
| AT1G27060 | - | 234/500 |
| AT1G27190 | + | 448/500 |
| AT1G27600 | - | 276/500 |
| AT1G28120 | - | 128/500 |
| AT1G28150 | - | 406/500 |
| AT1G29990 | - | 372/500 |
| AT1G30680 | - | 134/500 |
| AT1G30810 | - | 39/500 |
| AT1G32340 | - | 432/500 |
| AT1G33265 | + | 153/500 |
| AT1G34120 | - | 97/500 |
| AT1G42540 | - | 395/500 |
| AT1G42960 | - | 439/500 |
| AT1G44770 | + | 331/500 |
| AT1G47260 | + | 59/500 |
| AT1G48090 | - | 16/500 |
| AT1G48175 | - | 255/500 |
| AT1G48230 | - | 150/500 |
| AT1G48240 | - | 419/500 |
| AT1G49350 | + | 467/500 |
| AT1G50380 | - | 117/500 |
| AT1G50510 | - | 488/500 |
| AT1G50710 | - | 109/500 |
| AT1G51070 | - | 13/500 |
| AT1G53290 | - | 50/500 |
| AT1G54490 | + | 284/500 |
| AT1G54610 | - | 407/500 |
| AT1G54850 | + | 484/500 |
| AT1G55130 | + | 472/500 |
| AT1G55270 | + | 312/500 |
| AT1G55350 | - | 81/500 |
| AT1G55680 | + | 6/500 |
| AT1G56280 | + | 7/500 |
| AT1G58220 | + | 394/500 |
| AT1G59560 | - | 282/500 |
| AT1G59900 | - | 174/500 |
| AT1G60420 | + | 456/500 |
| AT1G60660 | - | 94/500 |
| AT1G60710 | - | 383/500 |
| AT1G61350 | + | 312/500 |
| AT1G62180 | + | 46/500 |
| AT1G62880 | + | 245/500 |
| AT1G63240 | + | 197/500 |
| AT1G63420 | + | 388/500 |
| AT1G63900 | + | 443/500 |
| AT1G64561 | - | 16/500 |
| AT1G65370 | + | 174/500 |
| AT1G65380 | - | 136/500 |
| AT1G65410 | + | 446/500 |
| AT1G65420 | + | 412/500 |
| AT1G66510 | + | 225/500 |
| AT1G67140 | - | 475/500 |
| AT1G67830 | + | 259/500 |
| AT1G69670 | - | 274/500 |
| AT1G70230 | + | 323/500 |
| AT1G70620 | + | 460/500 |
| AT1G71230 | + | 370/500 |
| AT1G71440 | + | 198/500 |
| AT1G71790 | - | 300/500 |
| AT1G72175 | - | 111/500 |
| AT1G73380 | + | 31/500 |
| AT1G73890 | + | 469/500 |
| AT1G73980 | + | 110/500 |
| AT1G74300 | - | 337/500 |
| AT1G74320 | + | 106/500 |
| AT1G74860 | - | 92/500 |
| AT1G74910 | - | 208/500 |
| AT1G75140 | - | 476/500 |
| AT1G75470 | - | 75/500 |
| AT1G75540 | + | 346/500 |
| AT1G75840 | + | 324/500 |
| AT1G76360 | - | 55/500 |
| AT1G77180 | - | 175/500 |
| AT1G77470 | - | 17/500 |
| AT1G78240 | + | 115/500 |
| AT1G78550 | - | 272/500 |
| AT1G78590 | + | 155/500 |
| AT1G79230 | - | 250/500 |
| AT1G79740 | - | 192/500 |
| AT1G79870 | - | 21/500 |
| AT1G80210 | + | 317/500 |
| AT1G80490 | - | 489/500 |
| AT2G02560 | + | 239/500 |
| AT2G02790 | - | 132/500 |
| AT2G03280 | + | 391/500 |
| AT2G04030 | + | 188/500 |
| AT2G04410 | + | 409/500 |
| AT2G04810 | + | 386/500 |
| AT2G04880 | - | 467/500 |
| AT2G06040 | + | 476/500 |
| AT2G11910 | + | 130/500 |
| AT2G13290 | - | 222/500 |
| AT2G13840 | - | 182/500 |
| AT2G14740 | - | 155/500 |
| AT2G15695 | + | 288/500 |
| AT2G17390 | + | 346/500 |
| AT2G18250 | + | 430/500 |
| AT2G20725 | + | 451/500 |
| AT2G21240 | - | 331/500 |
| AT2G21870 | + | 381/500 |
| AT2G23200 | - | 417/500 |
| AT2G23950 | - | 286/500 |
| AT2G24170 | - | 451/500 |
| AT2G24940 | + | 414/500 |
| AT2G25070 | - | 4/500 |
| AT2G25800 | - | 209/500 |
| AT2G25950 | + | 35/500 |
| AT2G26210 | - | 392/500 |
| AT2G26360 | - | 58/500 |
| AT2G26430 | - | 482/500 |
| AT2G26600 | + | 268/500 |
| AT2G26850 | - | 78/500 |
| AT2G27300 | + | 127/500 |
| AT2G27450 | + | 477/500 |
| AT2G28380 | - | 321/500 |
| AT2G29960 | + | 245/500 |
| AT2G30350 | + | 191/500 |
| AT2G30410 | + | 156/500 |
| AT2G30960 | - | 413/500 |
| AT2G31190 | + | 288/500 |
| AT2G31490 | - | 7/500 |
| AT2G31510 | + | 111/500 |
| AT2G31960 | - | 460/500 |
| AT2G34170 | - | 8/500 |
| AT2G34510 | + | 303/500 |
| AT2G34590 | + | 261/500 |
| AT2G34690 | + | 75/500 |
| AT2G35920 | + | 81/500 |
| AT2G36740 | - | 32/500 |
| AT2G38570 | + | 241/500 |
| AT2G39290 | + | 93/500 |
| AT2G40150 | - | 455/500 |
| AT2G40815 | + | 7/500 |
| AT2G41070 | - | 68/500 |
| AT2G41120 | - | 275/500 |
| AT2G41310 | - | 22/500 |
| AT2G42240 | + | 334/500 |
| AT2G42390 | - | 364/500 |
| AT2G42690 | - | 294/500 |
| AT2G43180 | - | 426/500 |
| AT2G43280 | - | 373/500 |
| AT2G43720 | + | 237/500 |
| AT2G43970 | - | 429/500 |
| AT2G44065 | + | 91/500 |
| AT2G44260 | + | 327/500 |
| AT2G44270 | + | 118/500 |
| AT2G45620 | + | 469/500 |
| AT2G46470 | + | 236/500 |
| AT2G46690 | + | 476/500 |
| AT2G47640 | + | 252/500 |
| AT3G01560 | - | 252/500 |
| AT3G01590 | + | 260/500 |
| AT3G03380 | - | 146/500 |
| AT3G03980 | + | 84/500 |
| AT3G04020 | + | 197/500 |
| AT3G05510 | - | 21/500 |
| AT3G05675 | + | 463/500 |
| AT3G05936 | - | 74/500 |
| AT3G06455 | - | 486/500 |
| AT3G06610 | + | 82/500 |
| AT3G06670 | - | 272/500 |
| AT3G07140 | + | 91/500 |
| AT3G07260 | + | 24/500 |
| AT3G08000 | + | 420/500 |
| AT3G08020 | - | 292/500 |
| AT3G09035 | + | 349/500 |
| AT3G09820 | + | 485/500 |
| AT3G10810 | + | 464/500 |
| AT3G10820 | - | 398/500 |
| AT3G11470 | + | 234/500 |
| AT3G11540 | - | 488/500 |
| AT3G11890 | - | 44/500 |
| AT3G12990 | + | 17/500 |
| AT3G13440 | - | 480/500 |
| AT3G13490 | - | 178/500 |
| AT3G13674 | + | 232/500 |
| AT3G15070 | + | 416/500 |
| AT3G15090 | - | 205/500 |
| AT3G15610 | - | 460/500 |
| AT3G15810 | - | 224/500 |
| AT3G17240 | - | 465/500 |
| AT3G17310 | - | 249/500 |
| AT3G17890 | - | 61/500 |
| AT3G18850 | - | 198/500 |
| AT3G20560 | + | 37/500 |
| AT3G20720 | - | 466/500 |
| AT3G21160 | - | 371/500 |
| AT3G22110 | - | 470/500 |
| AT3G22180 | + | 16/500 |
| AT3G22440 | - | 122/500 |
| AT3G22960 | + | 148/500 |
| AT3G23300 | - | 94/500 |
| AT3G23340 | - | 109/500 |
| AT3G23590 | + | 155/500 |
| AT3G24010 | + | 442/500 |
| AT3G24820 | - | 487/500 |
| AT3G25740 | + | 241/500 |
| AT3G26380 | - | 256/500 |
| AT3G26560 | + | 295/500 |
| AT3G26730 | + | 462/500 |
| AT3G26890 | - | 482/500 |
| AT3G27010 | + | 349/500 |
| AT3G27906 | + | 13/500 |
| AT3G42790 | - | 55/500 |
| AT3G43610 | - | 489/500 |
| AT3G46830 | - | 229/500 |
| AT3G47630 | + | 304/500 |
| AT3G48820 | + | 88/500 |
| AT3G49360 | - | 200/500 |
| AT3G50050 | - | 211/500 |
| AT3G51000 | + | 79/500 |
| AT3G51110 | + | 468/500 |
| AT3G52210 | + | 485/500 |
| AT3G52290 | + | 8/500 |
| AT3G52680 | + | 17/500 |
| AT3G52850 | - | 435/500 |
| AT3G53610 | - | 206/500 |
| AT3G53860 | + | 462/500 |
| AT3G54690 | - | 482/500 |
| AT3G55070 | - | 24/500 |
| AT3G55170 | + | 39/500 |
| AT3G55820 | - | 285/500 |
| AT3G56210 | - | 282/500 |
| AT3G56750 | + | 297/500 |
| AT3G57590 | - | 181/500 |
| AT3G58040 | - | 118/500 |
| AT3G58090 | + | 4/500 |
| AT3G59380 | + | 270/500 |
| AT3G60620 | - | 165/500 |
| AT3G61100 | + | 313/500 |
| AT3G61420 | + | 178/500 |
| AT3G61610 | - | 207/500 |
| AT3G61970 | + | 329/500 |
| AT3G62010 | - | 371/500 |
| AT3G63090 | + | 476/500 |
| AT3G63110 | + | 171/500 |
| AT4G00340 | - | 357/500 |
| AT4G00800 | - | 483/500 |
| AT4G00880 | + | 419/500 |
| AT4G01595 | + | 376/500 |
| AT4G01690 | + | 255/500 |
| AT4G01940 | + | 256/500 |
| AT4G02610 | - | 118/500 |
| AT4G03520 | + | 189/500 |
| AT4G04614 | - | 260/500 |
| AT4G04890 | + | 348/500 |
| AT4G05150 | - | 124/500 |
| AT4G08690 | - | 317/500 |
| AT4G08700 | - | 432/500 |
| AT4G10050 | - | 216/500 |
| AT4G10170 | - | 39/500 |
| AT4G10970 | + | 414/500 |
| AT4G11270 | + | 157/500 |
| AT4G11450 | + | 473/500 |
| AT4G13020 | - | 406/500 |
| AT4G14720 | - | 216/500 |
| AT4G14905 | - | 189/500 |
| AT4G14965 | + | 293/500 |
| AT4G15415 | - | 102/500 |
| AT4G16330 | - | 466/500 |
| AT4G16710 | - | 441/500 |
| AT4G16770 | - | 98/500 |
| AT4G16830 | - | 380/500 |
| AT4G17150 | - | 69/500 |
| AT4G17180 | - | 306/500 |
| AT4G17960 | - | 193/500 |
| AT4G18400 | - | 278/500 |
| AT4G18593 | + | 226/500 |
| AT4G18975 | + | 331/500 |
| AT4G19190 | - | 229/500 |
| AT4G19850 | + | 453/500 |
| AT4G20410 | - | 75/500 |
| AT4G22150 | + | 483/500 |
| AT4G22160 | - | 190/500 |
| AT4G22745 | - | 240/500 |
| AT4G23430 | + | 287/500 |
| AT4G23500 | - | 294/500 |
| AT4G24390 | - | 449/500 |
| AT4G25130 | - | 67/500 |
| AT4G26288 | + | 473/500 |
| AT4G26870 | + | 358/500 |
| AT4G27100 | + | 352/500 |
| AT4G27600 | - | 204/500 |
| AT4G27760 | + | 88/500 |
| AT4G28220 | + | 412/500 |
| AT4G28760 | + | 472/500 |
| AT4G28830 | + | 431/500 |
| AT4G29720 | - | 113/500 |
| AT4G31040 | - | 205/500 |
| AT4G31360 | + | 483/500 |
| AT4G31540 | + | 453/500 |
| AT4G32175 | + | 465/500 |
| AT4G32270 | + | 307/500 |
| AT4G32620 | + | 474/500 |
| AT4G32840 | + | 200/500 |
| AT4G32940 | - | 225/500 |
| AT4G32960 | + | 414/500 |
| AT4G33500 | + | 102/500 |
| AT4G34610 | - | 319/500 |
| AT4G34640 | - | 225/500 |
| AT4G34680 | + | 378/500 |
| AT4G35000 | + | 65/500 |
| AT4G35040 | + | 30/500 |
| AT4G35220 | - | 213/500 |
| AT4G35530 | - | 16/500 |
| AT4G35580 | + | 107/500 |
| AT4G35985 | + | 168/500 |
| AT4G36420 | + | 168/500 |
| AT4G37280 | - | 68/500 |
| AT4G38130 | - | 78/500 |
| AT4G38370 | + | 143/500 |
| AT4G39740 | - | 12/500 |
| AT4G40030 | - | 53/500 |
| AT4G40045 | + | 54/500 |
| AT5G01880 | + | 429/500 |
| AT5G03240 | - | 102/500 |
| AT5G03280 | - | 370/500 |
| AT5G04290 | - | 217/500 |
| AT5G04430 | + | 423/500 |
| AT5G04850 | + | 399/500 |
| AT5G05080 | + | 391/500 |
| AT5G05870 | + | 375/500 |
| AT5G07920 | - | 104/500 |
| AT5G08340 | - | 394/500 |
| AT5G08500 | + | 441/500 |
| AT5G08520 | + | 289/500 |
| AT5G09250 | - | 322/500 |
| AT5G10320 | + | 365/500 |
| AT5G10810 | - | 484/500 |
| AT5G11460 | + | 180/500 |
| AT5G11490 | + | 444/500 |
| AT5G12040 | - | 154/500 |
| AT5G12140 | - | 87/500 |
| AT5G12200 | + | 61/500 |
| AT5G13260 | + | 122/500 |
| AT5G14100 | + | 473/500 |
| AT5G14280 | - | 355/500 |
| AT5G14500 | + | 239/500 |
| AT5G14530 | - | 386/500 |
| AT5G14610 | + | 340/500 |
| AT5G14620 | - | 461/500 |
| AT5G15030 | + | 399/500 |
| AT5G16270 | - | 162/500 |
| AT5G16790 | + | 358/500 |
| AT5G16950 | + | 183/500 |
| AT5G17070 | + | 225/500 |
| AT5G17360 | - | 372/500 |
| AT5G17400 | + | 419/500 |
| AT5G19830 | + | 70/500 |
| AT5G20220 | - | 184/500 |
| AT5G20350 | - | 345/500 |
| AT5G20580 | - | 420/500 |
| AT5G20900 | + | 76/500 |
| AT5G23550 | - | 460/500 |
| AT5G23670 | - | 332/500 |
| AT5G24450 | + | 390/500 |
| AT5G26210 | + | 184/500 |
| AT5G27410 | + | 296/500 |
| AT5G28900 | - | 478/500 |
| AT5G35590 | - | 453/500 |
| AT5G36230 | + | 374/500 |
| AT5G37020 | - | 267/500 |
| AT5G39730 | - | 310/500 |
| AT5G40740 | + | 480/500 |
| AT5G40930 | + | 70/500 |
| AT5G41370 | - | 160/500 |
| AT5G41870 | + | 228/500 |
| AT5G41960 | - | 256/500 |
| AT5G41980 | - | 5/500 |
| AT5G42130 | - | 19/500 |
| AT5G42390 | - | 482/500 |
| AT5G42520 | + | 488/500 |
| AT5G42990 | - | 451/500 |
| AT5G43710 | + | 447/500 |
| AT5G43870 | - | 63/500 |
| AT5G44750 | + | 355/500 |
| AT5G46150 | - | 443/500 |
| AT5G46340 | + | 471/500 |
| AT5G47360 | + | 217/500 |
| AT5G47520 | + | 76/500 |
| AT5G47540 | + | 480/500 |
| AT5G47710 | - | 166/500 |
| AT5G47750 | - | 438/500 |
| AT5G47770 | - | 318/500 |
| AT5G49060 | - | 390/500 |
| AT5G49230 | + | 335/500 |
| AT5G49650 | + | 140/500 |
| AT5G49810 | + | 409/500 |
| AT5G49950 | - | 405/500 |
| AT5G53850 | - | 420/500 |
| AT5G54440 | - | 340/500 |
| AT5G54890 | + | 414/500 |
| AT5G55980 | - | 79/500 |
| AT5G56240 | + | 407/500 |
| AT5G56260 | - | 166/500 |
| AT5G56460 | + | 14/500 |
| AT5G56580 | + | 170/500 |
| AT5G56600 | - | 303/500 |
| AT5G56650 | - | 41/500 |
| AT5G56660 | + | 313/500 |
| AT5G57880 | - | 470/500 |
| AT5G58200 | + | 287/500 |
| AT5G58220 | + | 402/500 |
| AT5G58560 | - | 148/500 |
| AT5G59500 | + | 8/500 |
| AT5G60160 | + | 41/500 |
| AT5G60230 | - | 272/500 |
| AT5G60450 | + | 488/500 |
| AT5G61510 | + | 458/500 |
| AT5G62760 | - | 322/500 |
| AT5G62930 | - | 150/500 |
| AT5G63030 | + | 325/500 |
| AT5G63110 | - | 18/500 |
| AT5G63200 | - | 485/500 |
| AT5G63520 | + | 137/500 |
| AT5G63910 | - | 92/500 |
| AT5G64480 | + | 188/500 |
| AT5G64680 | + | 476/500 |
| AT5G65480 | + | 448/500 |
| AT5G65860 | + | 322/500 |
| AT5G66000 | + | 334/500 |
| AT5G66360 | - | 321/500 |
| AT5G67220 | + | 7/500 |
| AT5G67560 | - | 379/500 |

**\*** Overlapping sequences are merged to avoid redundancy. As a result some sequences in the final set may be longer than the specified maximum.

Distribution

+

-

Position values represent number of bases from the 5 prime end of the sequence provided to MEME and the length of that sequence. It is these figures expressed as proportions that are used for statistical analysis.

Probability value of comparison of these values to a linear distribution, p = 0.0019

The motif is found 243 times on the + stand and 239 times on the - strand.

Probability value that it is evenly distributed between the two strands, p = 0.45

Move the mouse pointer over the gene ids to highlight the corresponding marker
